# Comparison of 2D and 3D Plasma Electrolytic Oxidation (PEO)-Based Coating Porosity Data Obtained by X-ray Tomography Rendering and a Classical Metallographic Approach

**DOI:** 10.3390/ma15186315

**Published:** 2022-09-12

**Authors:** Polina Karlova, Maria Serdechnova, Carsten Blawert, Xiaopeng Lu, Marta Mohedano, Domonkos Tolnai, Berit Zeller-Plumhoff, Mikhail L. Zheludkevich

**Affiliations:** 1 Institute of Surface Science, Helmholtz-Zentrum Hereon, Max-Planck Strasse 1, 21502 Geesthacht, Germany; 2 Shenyang National Laboratory for Materials Science, Northeastern University, 3-11 Wenhua Road, Shenyang 110819, China; 3 Departamento de Ingeniería Química y de Materiales, Facultad de Ciencias Químicas, Universidad Complutense, 28040 Madrid, Spain; 4 Institute of Metallic Biomaterials, Helmholtz-Zentrum Hereon, Max-Planck Strasse 1, 21502 Geesthacht, Germany; 5 Institute of Materials Science, Faculty of Engineering, Kiel University, Kaiserstrasse 2, 24143 Kiel, Germany

**Keywords:** plasma electrolytic oxidation, pores, tomography

## Abstract

In this work, the porosity of plasma electrolytic oxidation (PEO)-based coatings on Al- and Mg-based substrates was studied by two imaging techniques—namely, SEM and computer microtomography. Two approaches for porosity determination were chosen; relatively simple and fast SEM surface and cross-sectional imaging was compared with X-ray micro computed tomography (microCT) rendering. Differences between 2D and 3D porosity were demonstrated and explained. A more compact PEO coating was found on the Al substrate, with a lower porosity compared to Mg substrates under the same processing parameters. Furthermore, huge pore clusters were detected with microCT. Overall, 2D surface porosity calculations did not show sufficient accuracy for them to become the recommended method for the exact evaluation of the porosity of PEO coatings; microCT is a more appropriate method for porosity evaluation compared to SEM imaging. Moreover, the advantage of 3D microCT images clearly lies in the detection of closed and open porosity, which are important for coating properties.

## 1. Introduction

Light alloys, such as Mg and Al alloys, are widely used in different industries where low density and good mechanical properties are required [1,2]. However, their poor corrosion resistance in aggressive environments demands surface modifications [2,3], including anodization [4], conversion [5], polymer layers [6], and—more recently—plasma electrolytic oxidation (PEO). 

PEO (also known as microarc oxidation—MAO) is an environmentally friendly electrochemical surface treatment process that produces relatively thick ceramic-like coatings under high voltages in various electrolytes (normally comprised of silicate, phosphate, and aluminate) [7,8]. This technique involves the polarization of the material up to high voltages (above dielectric breakdown), resulting in short-lived micro-discharges and the formation of a ceramic-like layer that is mainly formed by substrate- and electrolyte-derived constituents [9]. The basis of plasma electrolytic oxidation is that when a current is passed through the metal/electrolyte interface, under certain conditions, a high electric field strength is created that causes plasma micro-discharges at the electrode/electrolyte interface [10]. 

The result of the action of plasma micro-discharges is the formation of a coating layer consisting of oxidized forms of substrate metal elements and electrolyte components. Depending on the PEO method and the composition of the electrolyte, it is possible to obtain ceramic coatings with unique characteristics and a wide range of practical applications [2,10,11,12]. For example, PEO is known as a barrier coating for corrosion protection applications (playing the role of a barrier layer between the substrate and corrosive environment [13,14]). The hardness of the ceramic coatings also improves the wear properties of metal surfaces [11], e.g., Li et al. [15] demonstrated a wear volume loss decrease of 10 times.

However, these plasma discharges are also responsible for the formation of defects in the coating, such as the remains of discharge channels, pores, and cracks [7]. This porosity strongly compromises the coating’s corrosion protection properties as aggressive electrolytes can directly penetrate through the pores, reaching the substrate interface and causing corrosion processes [16]. On the other hand, these pores can be considered potential micro-containers, which can be loaded with functional molecules or nanocontainers [16,17,18,19] to create a multifunctional coating. Additionally, the pores can play a bio-physical anchor role, facilitating the integration of the material, when talking about PEO-treated implant materials and their biocompatibility with bones [17,20].

There are several factors that have been reported to influence the porosity of the PEO layer, such as the substrate material, treatment time, or the type of power supply or electrolyte. Rakoch et al. [21] studied the influence of alloy composition on the PEO formation process for two different magnesium-based cast alloys; it was demonstrated that even a relatively small change in the alloy (around 6% additional aluminium) significantly changes the mechanism of the PEO coating formation. In [22], the authors reported that porosity and pore size depend on the treatment time and thus on the thickness of the coating. Longer treatment times result in higher porosity and thickness and a larger population of large-sized open pores. Electrical parameters affect the stability of sparks during processing, and this affects the porosity of the final coating, e.g., Quintero et al. reported an influence of the operation mode (galvanostatic and galvanostatic–potentiostatic), current density, and potential [23]. Alternating current (AC) power supplies allow one to obtain compact coatings with fewer defects [24]. A reversal of sample polarity causes a discharge interruption, which precludes the formation of long-lasting, very massive micro-discharges, resulting in the formation of smaller pores [25]. The electrolyte composition’s influence was demonstrated in work [26]; the authors reported a higher porosity in the case of phosphate and silicate electrolytes when compared to aluminate electrolytes. The addition of particles to the electrolyte may further change the number of pores and their size [22]. Particles may also change the electrical properties of the PEO layer due to the melting, reaction, and formation of new phases (reactive incorporation), or may be inertly incorporated into the coating [27].

X-ray micro computed tomography (microCT) is a promising method for the analysis of pore shape and distribution in PEO coatings. MicroCT is a non-destructive 3D imaging technique that converts a series of X-ray radiographs into a volumetric representation of the sample. It allows one to evaluate the size and forms of pores, which is not available (or is limited) by standard methods such as light or electron microscopy [28]. However, the equipment for microCT is not so easily accessible—unlike microscopy—and there are several limitations for tomography based on the sample size and material, which can decrease the efficiency of the method. The main limitation of classical microscopy is the impossibility of the 3D characterization of complex pore cluster structures. The only option is to use focused ion beam (FIB)-based scanning electron microscopy (SEM) to look at the structure layer-by-layer and take a picture at each step. Cross-sectional microscopy is typically a destructive method and is much slower than the microCT approach. 

There are not many studies where microCT has been used for the evaluation of the microstructure of PEO coatings. In the work of Lu et al. [22], the evolution and distribution of the pores and particles incorporated in a PEO coating on an AM50 Mg alloy was studied. It was shown that open pores could potentially be a large defect that is not visible from the surface. Zhang, X. et al. demonstrated in their work [28] that microCT can be used to detect pores from 6 μm^3^ using a Zeiss Xradia Versa 520 lab-based X-ray CT scanner. In the current study, the limitations and applicability range of surface microscopy, cross-sectional microscopy, and computer tomography for the determination of the porosity of PEO coatings are investigated for three different substrate materials. The analysis of the obtained pore size distributions is performed for the same coatings using different methods. For the supplementary characterization of the resulting PEO layers, additional techniques such as XRD and SEM were applied.

## 2. Experimental Part

### 2.1. Materials

**Figure 1:**
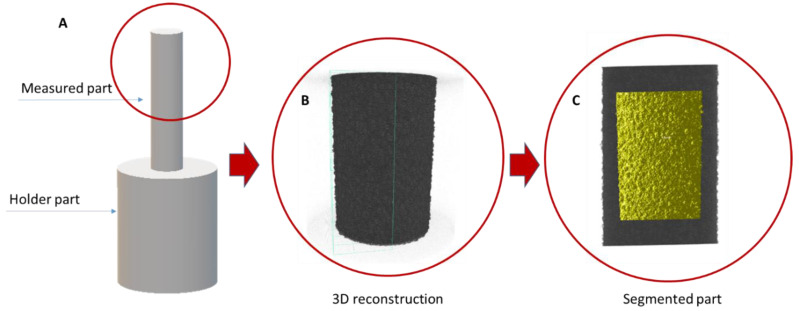
(**A**). Scheme of the sample’s geometry; (**B**). X-Ray CT data rendered in 3D; (**C**). Highlighted area shows representative volume element.

Three cylindrical pin-shaped specimens (Figure 1A) were lathed from AZ31, AZ61, and AlMg3 light metal alloys. The nominal elemental composition was determined by arc spark optical emission spectroscopy (Spectrolab M9, Ametek-Spectro, Germany; Table 1).

### 2.2. PEO Treatment

AC PEO treatment was conducted for 600 s using a PE Pulse Reverse Power Supply pe861UA-500 (plating electronic GmbH, Sexau, Germany) rated at max. 500 V/24 A. The specimens (Figure 1A) were immersed in an alkaline silicate solution (2 g/L KOH + 10 g/L Na_2_SiO_3_) and constant voltage pulses were applied to them. The positive-to-negative pulse ratio was 420/−30 V at a 500 Hz frequency. The PEO process was performed at 10 °C ± 2 °C. After PEO processing, the specimens were rinsed in distilled water and dried in warm air.

### 2.3. Methods

A Bruker D8 Advance X-ray diffractometer (Karlsruhe, Germany) was used for the phase composition analyses. The measurements were carried out using Cu-Kα radiation in the range of 2 theta from 20° to 80° (exposure time 1 s, step 0.02°) under a 3° incident angle. 

Plan views and cross-sections of coatings were examined with a Tescan Vega3 SB scanning electron microscope (SEM, Brno, Czech Republic) using backscattered electron mode (BSE). The coating composition was determined by energy dispersive X-ray spectrometer (EDS) analysis (Heidenrod, Germany). 

The specimens were embedded in resin and cross-sections vertical to the pin axis were prepared by grinding through successive grades of silicon carbide paper, with final polishing up to 1 μm diamond finish.

The synchrotron radiation microtomography experiments were conducted at the P05 end station of a Petra III, (DESY, Hamburg, Germany) at an energy of 18 keV. Only the upper cylindrical pin part, with an average diameter of 1.2 mm and 6 mm in length, was measured. The distance between the sample and the detector was 15 mm. During the acquisition of the tomograms, 1800 projections were taken—with an acquisition time of 3 s each. The reconstructions have dimensions of 960×960×1528 voxels, with a voxel size of 1.1 μm^3^.

### 2.4. Computational Approach

The main idea of segmentation in the case of PEO coatings is to define the difference between the oxidized layer, air (pores), and substrate. Segmentation was carried out with a global thresholding and a subsequent growth of the segmented regions based on the grey levels of the connected voxels. Finally, manual corrections were undertaken on segmented regions where the accuracy was not high enough for the quantitative analysis.

To simplify the characterization and discussion, a sample segment with a height of 1.2 mm—1/4 of the initial cylinder circle—was considered to be a representative volume element, as indicated in yellow in Figure 1C. Each tomography dataset was cropped and had the dimensions 751 × 822 × 1092 voxels. Pores less than 27 voxels in volume (3 µm pore diameter) were excluded from the evaluation in order to avoid errors from the size effect on the morphological description of the segmented dataset.

Dragonfly software ver. 2020 (Object Research Systems (ORS), Montréal, Québec, Canada) was applied for tomography data processing as well as the quantitative characterization of porosity (2D and 3D) using the following equation:

(1) $$P=\frac{Vp}{Vp+Vpeo}\times 100\%$$

where P is the porosity, *Vp*—volume of pores (both open and close), and *Vpeo*—volume of PEO layer.

Avizo™ Fire 9.3.0 software (Thermo Fisher Scientific Inc, Waltham, MA, USA) was applied for the evaluation of the morphological parameters of the pores.

There are several imaging methods for estimating porosity (the sum of the open and closed porosities): from the surface or from cross-section images. One of the most widely available non-destructive techniques is the calculation of the porosity percentage on the surface of the PEO coatings (2D), when visible-from-surface voids are assumed to be pores (based on contrast) and the area of the voids is compared with the total analysed area [23]. However, the surface analysis only allows one to consider the open pores of the layer. In order to also get access to the closed pores, the cross-sectional morphology should also be investigated. In this work, 3 images of 401 × 401 pixels of each sample’s surface were studied to determine the 2D surface porosity and images of 1024 × 404 pixels were used for the 2D cross-section porosity.

## 3. Results and Discussion

### 3.1. Basic Coating Characterization

**Figure 2:**
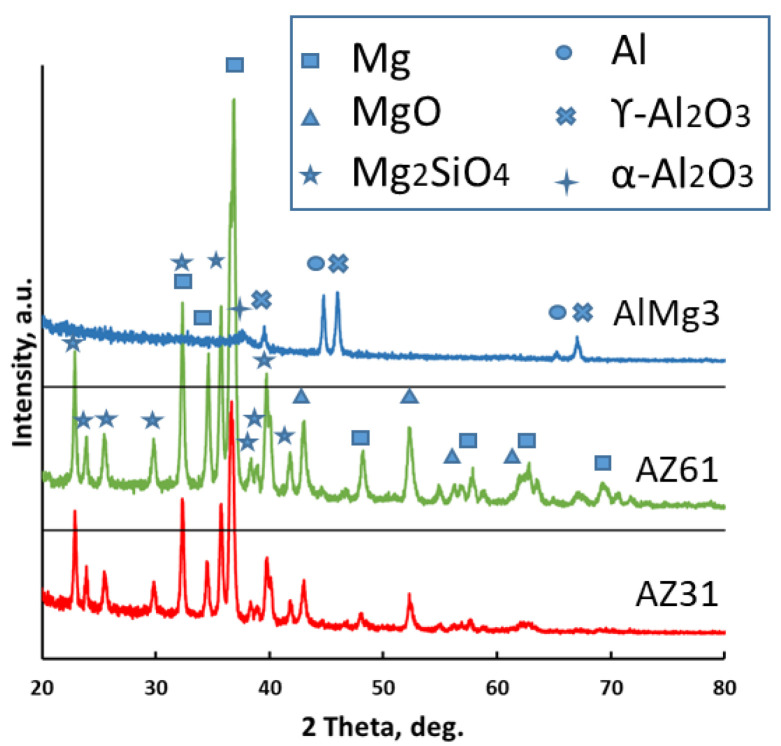
XRD patterns of the final PEO coatings on the AZ31, AZ61, and AlMg3 samples.

Figure 2 displays the X-ray diffraction patterns of the formed PEO coatings. The XRD examination depicted high intensity peaks for Mg (for the AZ31 and AZ61 alloys) and Al (for the AlMg3 alloy), originating from the base material as the coatings were penetrated by the X-rays. 

The PEO coatings produced on AZ31 and AZ61 were predominantly composed of two crystalline phases: Mg_2_SiO_4_ and MgO. In the case of the PEO layer on the AlMg3, the XRD analysis revealed that the coating was mainly composed of α-Al_2_O_3_ and γ-Al_2_O_3_. 

**Figure 3:**
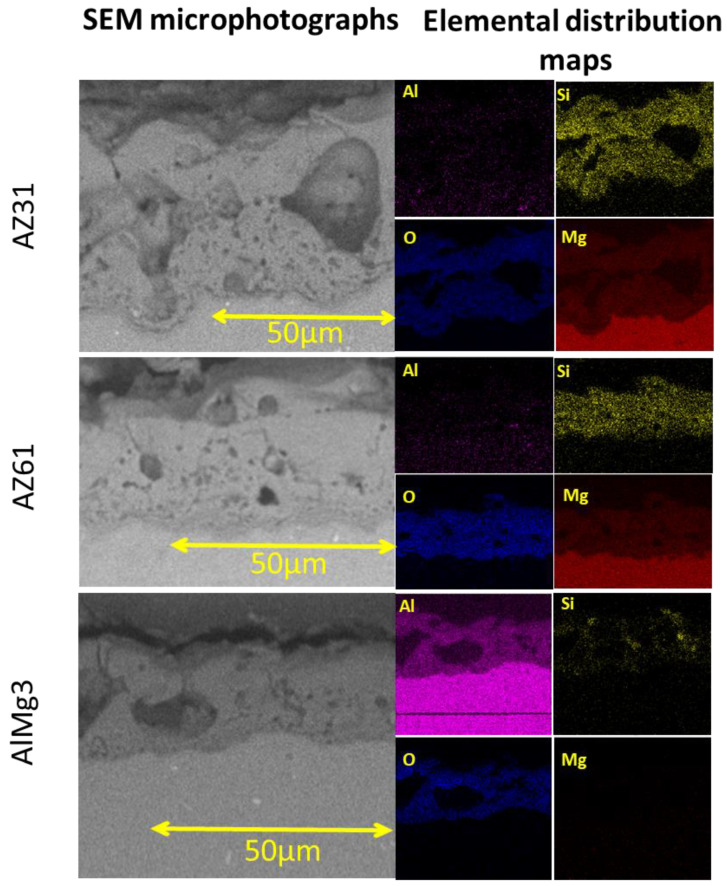
Cross-sectional EDS of the AZ31, AZ61, AlMg3 samples.

In order to supplement the information on the chemical composition, EDS elemental mapping of cross-sections was conducted (Figure 3); the analysis results are displayed in Table 2.

For Mg-based (AZ31 and AZ61) samples, the EDS results correlate with the XRD data. The presence of relatively uniform distributed Si in the coating is visible and confirms the presence of a Mg_2_SiO_4_ phase. Although for AlMg3 there was some silicon detected in the PEO layer, there were no corresponding reflections visible in the XRD patterns; this can be explained via the formation of an amorphous phase containing silicon [29].

**Figure 4:**
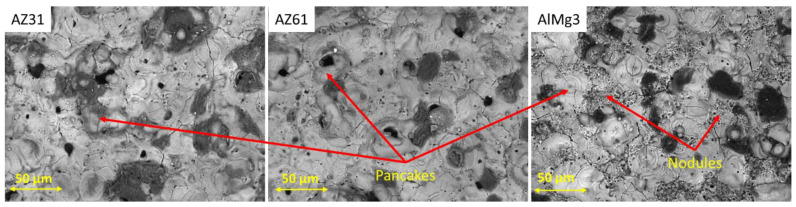
SEM images of the PEO coating surfaces.

SEM images of all the obtained PEO coatings were taken in order to describe the surface morphology of the layers (Figure 4). A typical PEO morphology can be observed, where there are randomly distributed pores and cracks in the coating surface, which indicate a non-uniform defect distribution in the coating volume.

In [30], the authors described the surface morphology of PEO layers as nodules and pancakes, where a pancake structure is more typical for strong micro-discharges. In Figure 3, one can see that these pancakes are more representative of Mg-based alloys. On the other hand, a combination nodule and pancake structure is more specific for the Al-based alloy. These nodules have many micro-pores and, according to [31], appear due to cracks and pores full of electrolytes in the initial stages of the PEO formation. Assuming a comparable energy input during PEO processing, the differences in surface morphology for the coatings on the Al and Mg substrates can most likely be associated with the different melting temperatures (T_M_) of the formed phases on the respective surfaces (2040 °C for ɤ-Al_2_O_3_, 2852 °C for MgO, 1710 °C for SiO_2_, and 1890 °C for Mg_2_SiO_4_). One can see that forsterite, a main PEO phase on the Mg substrates, has a lower melting temperature in comparison with MgO and ɤ-Al_2_O_3_. When SiO_2_ is melted, it can firstly react with solid MgO or Mg(OH)_2_, stimulating faster liquid sintering and forming the forsterite. This results in the simpler local formation and larger volume of liquid phase on Mg in comparison to Al, due to the plasma discharges. The lower viscosity of the molten forsterite phase also explains why there is more melt pushed out of the discharge channel compared to the molten Al_2_O_3_; both form pancake structures, but the Mg more often has a central open discharge channel (pore), compared to the more closed ones formed with Al. Additionally, the higher thermal conductivity of Al plays an additional role, contributing to lower and shorter peak temperatures after discharges. Overall, the melt pools remain smaller on the Al, and there are nodular-looking areas that remain around the strong discharges where solid-state sintering seems to continue to dominate. 

### 3.2. Analysis of X-ray CT Images

**Figure 5:**
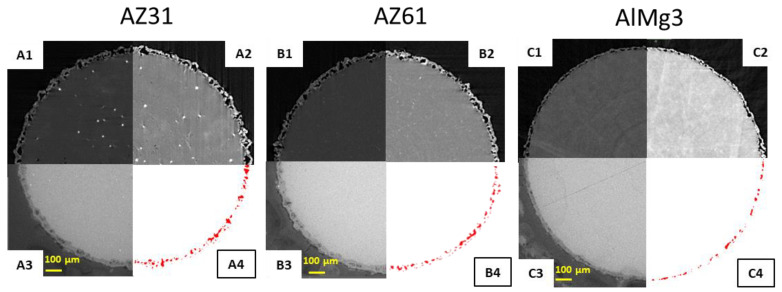
(**A1**–**C1**) X-ray microCT raw images after reconstruction; (**A2**–**C2**) X-ray microCT images with adjusted brightness and contrast; (**A3**–**C3**) SEM images; (**A4**–**C4**) segmented pores.

X-ray microCT was conducted for each sample in order to evaluate the porosity of the PEO coatings. The contrast of microCT images depends directly on the X-ray attenuation coefficients of the materials. Unfortunately, at an energy of 18 keV, the values of the attenuation coefficients are very close and cannot provide significant differences in contrast between the coating and substrate [32]. MicroCT images with different brightness and contrast combined with SEM images and reconstructed 2D images of pores are shown in Figure 5.

**Figure 6:**
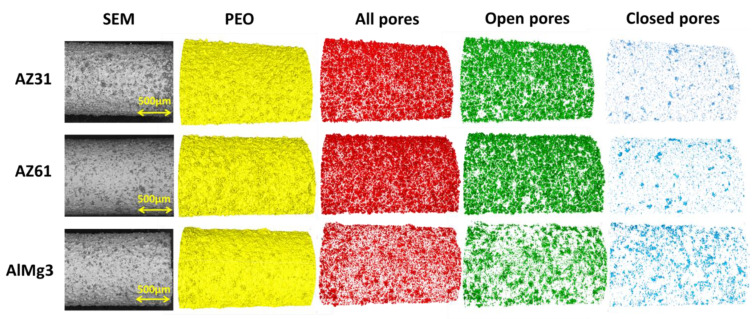
Comparison of results of the X-ray CT data processing for the AZ31, AZ61, and AlMg3 alloys.

In Figure 5(A1–C1), one can see the low contrast between the coating and substrate and, as was mentioned above, it can be explained by the settings of the visualization and the relatively low difference in the X-ray attenuation coefficients of the phases (substrate, coating). However, the brightness and contrast could be adjusted manually (Figure 5(A2–C2)). With additional data processing, pores could be “extracted” from CT images (Figure 5(A3–C3)) and analysed. The 3D reconstruction of pores and PEO could be carried out after processing of the initial CT datasets and the selection of different regions of interests (PEO, pores, and substrate; Figure 6).

A comparison of SEM images of the initial samples with the microCT rendering of the surface (Figure 5 and Figure 6) shows an adequate level of resemblance between them. 

Within the current work, the pores that have no detected connection to the outer environment are called “closed” pores. Pores with an identified connection to the outer environment are called “open” pores. For Mg-based samples (Figure 6), open pores were mostly represented as large “spherical” pores, and closed pores were generally smaller. On the contrary, in the AlMg3 sample, one can see fewer pores in total and the closed pores look bigger on average than in the Mg-based samples. It is worth mentioning that there are several micron and sub-micron sized pores less than 29.7 µm^3^ that cannot be detected by microCT or that are excluded from evaluation due to its resolution limit based on voxel size and optics [28]. 

**Figure 7:**
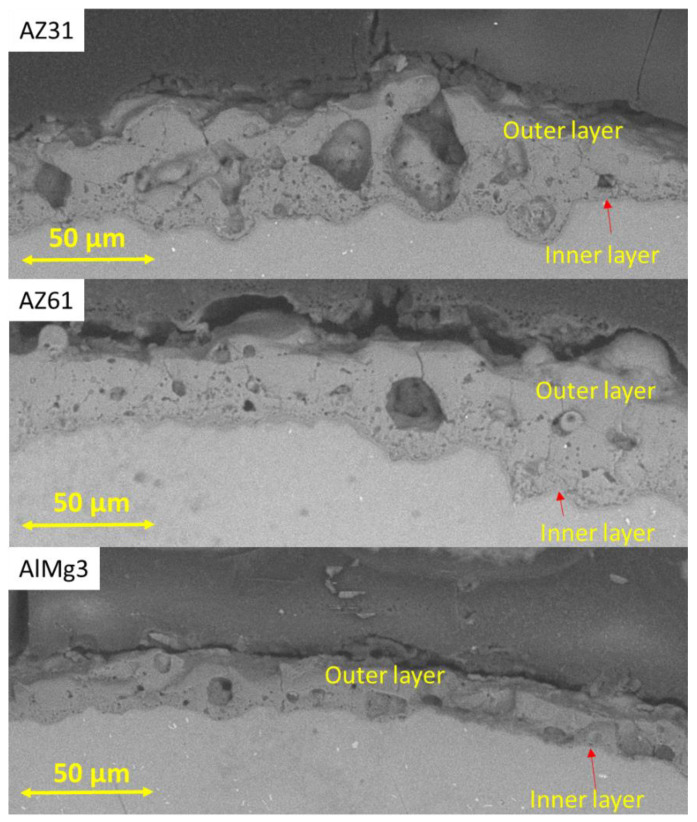
Cross-section SEM images of the AZ31, AZ61, and AlMg3 samples.

PEO coatings often have a layered structure; so, to evaluate the distribution of pores in the layers, additional cross-section images were taken (Figure 7). However, at short treatment times, these clearly separated layered structures were still not formed, and the dense inner layers were still missing—except the inner barrier layer.

One can see that the big pores were mainly located in a central pore band of the PEO layer, which is typical for such coatings [25]. This divides the coating into an outer, more densely sintered part and an inner part. This can be explained by the higher amount of high-energy micro-discharges in the vertical pores. This also results in a relatively thin inner layer; due to the high amount of energy released during the ignition in the spherical pores, there is less energy “available” for the growth of the inner layer. For Mg samples, small, closed pores were mainly located in the inner layer—while for the Al sample, they were located in both the inner and outer layers. In addition, the thickness of the final PEO coating on the Al sample was lower than in the Mg samples. The pores in the inner barrier layer are not distinguishable by SEM analysis as they are significantly smaller in comparison with those in the outer layers [2].

### 3.3. Calculation of Porosity

**Figure 8:**
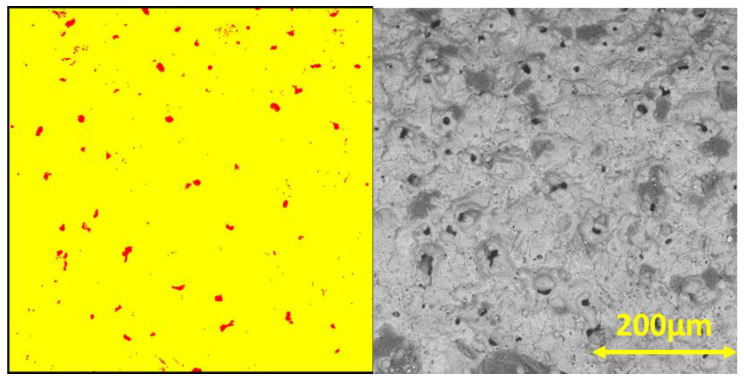
Model of 2D surface porosity and initial SEM image.

One of the examples of such a visualization for the 2D porosity calculation used within the current paper is shown in Figure 8 (an example of the AZ31 alloy is given). 

The 2D surface porosity measurements were conducted in three different areas of each sample and had the names “porosity 1”, “porosity 2”, and “porosity 3”, respectively, in Table 3.

One can see that the values of 2D porosity in samples AZ31 and AZ61 were quite close, and that they were higher than the calculated porosity of the AlMg3. It is worth it to mention that 2D surface porosity varies with the position of the area of the sample and that the statistics depend on the size of the measured and studied areas. Even though three measurements could not be rated as statistically correct, we assume that this was sufficient to demonstrate the disadvantages of this method. 

**Figure 9:**
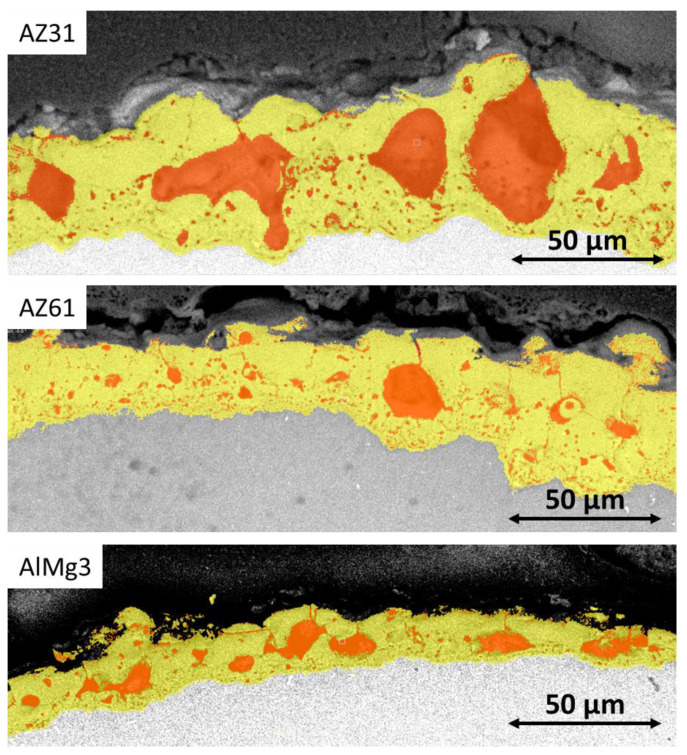
Model of the 2D cross-sectional porosity of the PEO coatings on the three selected substrates: Red—pores, yellow—PEO coating.

However, 2D surface analysis does not take into account the presence of closed pores, cracks, and voids, which can be seen on cross-section images. Cross-section images are typically more widely available compared to tomography techniques. However, this method of porosity evaluation also has similar disadvantages to tomography, based on the resolution and contrast of final images. In addition, cross-section preparation is a destructive method. Another way to calculate 2D porosity is based on cross-section images (Figure 9).

The results of the 2D porosity cross-section calculations for the three selected coatings are also represented in Table 3. The large difference between the 2D surface and 2D cross-sectional porosity values demonstrates that 2D surface porosity is a less reliable method for the evaluation of PEO coating porosity due to the high impact of closed pores and pore form factor on calculation results. 

The results of the 2D cross-sectional porosity demonstrate significant differences between the porosity of AZ31 and AZ61, which does not correlate with the 2D surface porosity data or the pore distribution presented in Figure 6. To identify whether any of these methods demonstrate satisfactory accuracy for the porosity of PEO, evaluation calculations were carried out using the microCT data (Table 4). The results are presented for the area indicated in Figure 1C.

As is demonstrated in Table 4, the difference in porosity between the two Mg-based samples is small. However, the level of porosity strongly depends on the substrate material. The percentage of closed pores inside the PEO coating on the Mg-based samples is lower than in the Al-based sample; at the same time, the volume and percentage of open pores in the AlMg3 is notably lower than in both the Mg-based specimens. It is worth mentioning that open pores make the largest contribution to porosity. In addition, the ratio between the open and closed pores is higher in the AZ31 and AZ61 than in the AlMg3. One can also observe that the total porosity of the PEO layer on the Al-based sample is almost two times lower.

**Figure 10:**
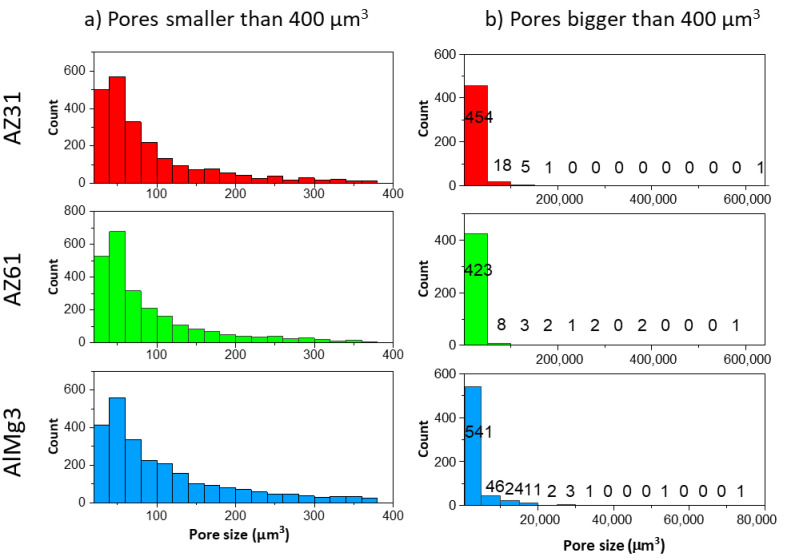
Histogram of pore size distribution: (**a**) pores smaller than 400 µm^3^; (**b**) pores bigger than 400 µm^3^.

The 2D surface porosity measurements show highly underestimated values compared to the 3D microCT results, but the fact that there are more pores in the Mg-based samples is correctly represented by the 2D surface calculation as well. The comparison of the 2D cross-section calculations and microCT results demonstrates that single-image processing is not sufficient to produce reliable data. To improve the quality of this method, a set of cross-section images comparable with the tomography dataset would need to be processed, which requires excessive amounts of time compared to tomography. However, the initial tomography data (set of projections), in principle, is similar to the set of cross-section images (Figure 5(A1–A3,B1–B3,C1–C3)). It also allows one to obtain more statistical data, such as pore size distribution, which is important to understand the coating’s properties. The data, obtained by Avizo software, is presented in Figure 10. 

One can see that both very small (smaller than 100 µm^3^) and very big (larger than 20,000 µm^3^) pores were detected in the coatings. Besides this, all three samples showed similar trends in pore distribution in the volume range up to 400 µm^3^. The main differences were in the formation of huge pores. More statistical data is available in Table 5.

**Figure 11:**
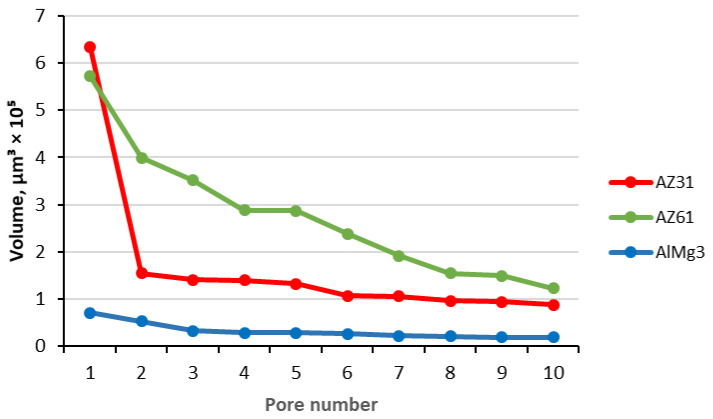
Volume of the 10 biggest pores (µm^3^).

The AlMg3 sample displayed a smaller deviation in pore size compared to both the Mg samples. At the same time, 80% of the pores were in the range 28–388 µm^3^ for the AlMg3, while for the AZ31 and AZ61, this range was significantly smaller. In addition, the biggest pore in the AlMg3 was almost nine-times smaller than in the AZ31. A comparison of the size of the 10 biggest pores in the samples is shown in Figure 11.

**Figure 12:**
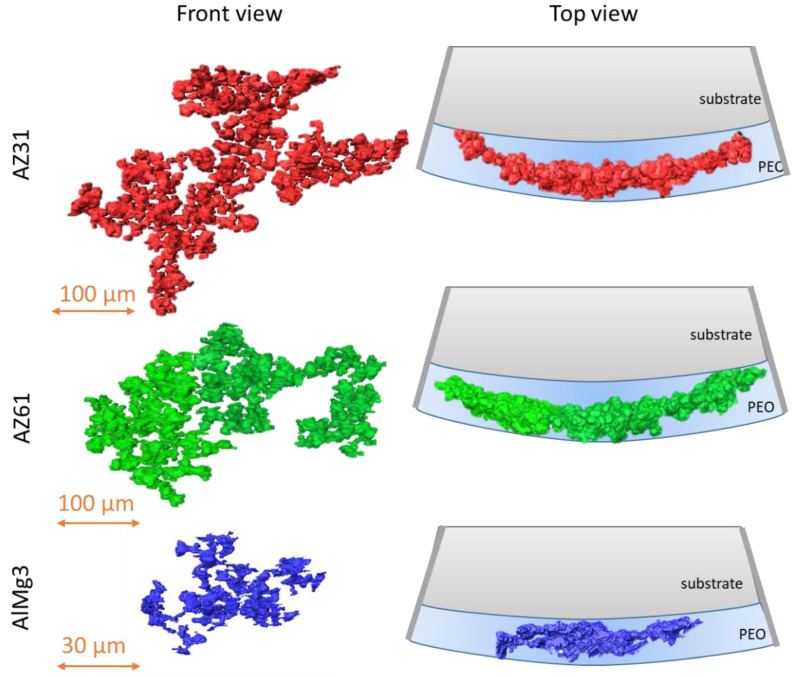
Pore morphology in AZ31; AZ61; and AlMg3.

A big difference in the substrate’s influence on the formation of large pores is visible. The biggest pores in the AlMg3 were much smaller than in the AZ31 and AZ61. This data, in combination with Table 5, demonstrate a great contribution of single huge pores within Mg-based samples to total porosity values. However, these huge pores are actually clusters of smaller pores, which one can see in Figure 12.

**Figure 13:**
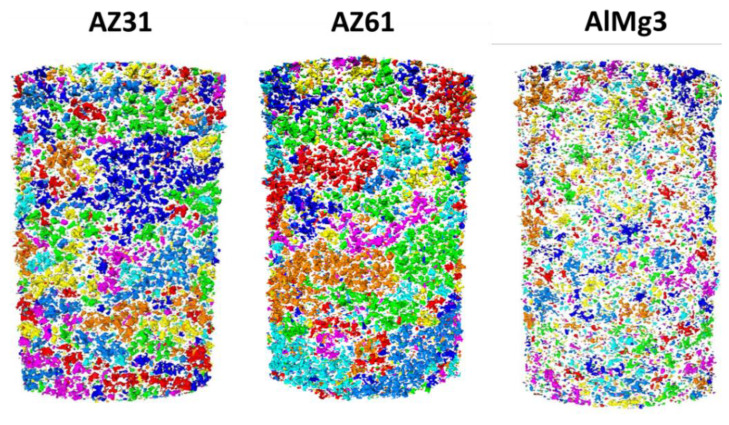
Pore cluster distribution colour map.

Clusters can be described as spherical pores connected with channels. Such a system cannot be detected by SEM surface and cross-sectional imaging due to the high complexity of the structure and its location inside the PEO coating. These huge pores connect the atmosphere with the substrate through the PEO layer. These connections can significantly compromise the corrosion properties of the PEO coating as aggressive electrolytes can directly reach the interface. In Figure 13, one can see distribution of pore clusters in the 3D model, where each colour shows one big pore; however, due to the limited number of colours, they may be repeated.

A large number of pore clusters could be observed in each sample. At the same time, the cluster distributions in the Mg-based samples were similar. The PEO coating on the Mg substrate demonstrates a trend to form bigger pores clusters compared to the Al substrate. 

## 4. Discussion

At first glance, the surface SEM pore analysis looks to be the fastest and easiest way to analyse pores in the PEO layer on various substrates. However, this analysis does not allow one to detect closed pores at all, and the shape of pores under the surface is also not visible. From this point, such a method is the least suitable for exact porosity evaluation. Nevertheless, it is the fastest and quickest approach to estimate quality in terms of corrosion resistance—if the treatment time is not long (<10 min). Within short times, a dense inner layer is normally not developed and open outer pores go directly to the barrier layer at the interface. 

As a more complex approach, the analysis of SEM cross-sections can be suggested for pore analysis. However, it is not possible to determine whether pores are closed by only analysing the SEM cross-section images. SEM images only allow one to suspect the presence of closed pores. The number and volume of pores cannot be accurately evaluated using SEM images due to their complex morphology. However, the actual values in this study allowed a reasonable estimation, with not too much difference to the actual values.

The porosity of PEO coatings can be measured not only with imaging methods, but also with methods based on the penetration of different substances (liquids, gases) into a porous structure. For instance, in work [33], the apparent density method, helium pycnometry, isothermal nitrogen absorption (BET), and mercury intrusion porosimetry were used to evaluate the porosity of a silicate-based PEO coating on an Al substrate. The porosity value was reported to be ~20% for the BET and mercury intrusion porosimetry. However, the comparison of skeletal density using different penetration fluids (namely helium, mercury and C_11_F_20_ liquid) resulted in a variation of porosity values between 4 and 40%. This difference could be associated with the viscosity of the liquid and its ability to penetrate into relatively small pores. Although penetration methods and surface imaging do not presume the presence or high impact of closed pores on total porosity, the authors used these methods, since it had been shown that the closed porosity was low. Such an approach can be used when the properties of the PEO coating are determined by its accessible surface area, e.g., for photocatalytic applications. 

The X-ray microCT results allowed us to separate the contribution of closed porosity from the total porosity of the layer. This knowledge could be crucial in the case of, e.g., wear analysis of the layer, when the overall mechanical properties of the coating are important. On the other hand, this approach is the most time and computational resource-consuming and can be applied only in strongly justified cases. In the frame of the current study, the comparison of computer tomography vs. SEM surface and cross-sectional imaging was performed. It was shown that, in the case of Mg-based samples, the level of closed porosity was negligible—while for the AlMg3 sample the closed porosity could not be ignored, which was interestingly already indicated by the observation of the surface morphology showing a large number of closed pores on the surface (Figure 4). Besides this, none of the metallographic imaging methods mentioned above is able to detect and evaluate the presence of huge pore agglomerations, which may have a dramatic influence on the corrosion properties of PEO-coated samples. In other words, porosity is a volume characteristic—this is why microCT is a preferable method for porosity evaluation, due to the larger amount of data obtained after non-destructive analysis and 3D characterization. Simple surface and cross-sectional image analysis does not provide reasonably valuable data sets for such an analysis.

In order to study the influence of a substrate material on PEO coating, Mg- (AZ31 and AZ61) and Al-based (AlMg3) alloys were used. As was mentioned above, PEO coating on the Al substrate demonstrated lower porosity compared to coatings on Mg substrates, as well as a lower coating thickness. This can be explained by the fact that Al has a much higher thermal conductivity than Mg, which allows the amount of melted material at the point of micro-discharges during the PEO coating formation to be decreased, resulting in smaller pore sizes. The higher heat generated by the discharges also forms a thicker layer [34]. Moreover, the relatively high melting temperature of ɤ-Al_2_O_3_, forming the PEO layer on Al, in comparison with Mg_2_SiO_4_—presenting in the PEO layer on Mg alloys—requires a higher energy input during the treatment for equivalent effective liquid phase sintering. Since the treatment time and voltage parameters were selected to be constant for the characterized coatings, the PEO on the Al was thinner in the end. The addition of potassium fluoride to the electrolyte reduces the total porosity of magnesium-based samples due to the formation of a fluoride-reach dielectric layer on the surface [35]. In addition to the different average thickness of the coating, the various melting temperatures of the compounds forming the PEO layer can also result in the differing porosity of the coating. In the case of a lower T_M_, a higher amount of liquid phase is produced in the plasma discharge. This liquid phase moves out of the substrate interface and solidifies, facing the electrolyte on the surface. Due to the higher volume of melted phases in the case of a lower T_M_, the larger remaining pores form the PEO layer. Another possible explanation for the difference in the thickness of the PEO layers on the Mg and Al is that the oxidation of Al is less favourable compared to Mg (up to ~1550K), according to the Ellingham diagrams [36]. 

## 5. Conclusions

Based on microCT, SEM images, and complementary XRD and EDS analyses of silicate-based PEO coatings on AZ31, AZ61, and AlMg3 alloys, the following conclusions were made:

The 2D surface porosity calculations did not demonstrate sufficient accuracy to be a recommended method for the exact evaluation of the porosity of PEO coatings. However, it is the quickest approach and is still justified for thin coatings (with short treatment times) when no dense inner layer is formed and open discharge channels have direct contact with the barrier layer. 

MicroCT is a more appropriate method for porosity evaluation compared to SEM imaging. In particular, it can reveal the connection between the pores and the complex agglomerated pore structure. 

PEO coating total porosity is mostly represented by open pores. However, there is a difference between aluminium and magnesium; the latter shows porosity that is more open. 

Pores on Mg-based samples show a trend of being more open and forming large agglomerations. They connect the environment with substrate, which can have negative effect on the corrosion resistance of the layers.

## Figures and Tables

**Table 1:** The elemental composition of the alloys.

Alloy	Chemical Composition (wt. %)								
	Al	Zn	Mn	Si	Fe	Be	Mg	Cu	Ti
AZ31	3.04	1.00	0.27	0.02	0.0028	0.00046	Balance	0.0008	-
AZ61	5.5	1.5	0.78	0.02	0.004	0.00013	Balance	0.0006	-
AlMg3	Balance	-	-	0.07	0.07	-	2.86	0.01	0.01

**Table 2:** Elemental composition of the PEO coatings.

Sample	Mg	Al	O	Si	Zn
AZ31	24.7 at.%	0.68 at.%	55.49 at.%	18.97 at.%	0.15 at.%
AZ61	26.26 at.%	0.68 at.%	55.99 at.%	17.01 at.%	0.07 at.%
AlMg3	0.47 at.%	25.58 at.%	57.60 at.%	16.30 at.%	>0.01 at.%

**Table 3:** Results of the 2D investigation of open porosity from coating surface (2D) and cross-section (2D-CS) images.

Sample	2D Porosity 1	2D Porosity 2	2D Porosity 3	2D Average	2D Deviation	2D-CS Porosity 1	2D-CSPorosity 2	2D-CSPorosity 3	2D-CS Average	2D-CS Deviation
AZ31	1.59%	3.13%	3.72%	2.81%	0.82%	27.88%	19.60%	33.22%	26.90%	4.87%
AZ61	3.34%	1.38%	3.34%	2.69%	0.87%	14.42%	17.99%	15.84%	16.08%	1.27%
AlMg3	0.82%	1.32%	1.55%	1.23%	0.27%	19.51%	16.25%	31.65%	22.47%	6.12%

**Table 4:** Results of the microCT pore characterization.

Parameter	AZ31	AZ61	AlMg3
Volume of closed pores	0.037 mm^3^	0.046 mm^3^	0.073 mm^3^
Volume of open pores	0.656 mm^3^	0.624 mm^3^	0.185 mm^3^
Volume of PEO	2.381 mm^3^	2.336 mm^3^	1.714 mm^3^
Porosity of PEO layer (closed pores)	1.21%	1.52%	3.68%
Porosity of PEO layer (open pores)	21.34%	20.77%	9.39%
**Total porosity**	**22.55%**	**22.29%**	**13.07%**

**Table 5:** Statistical information on pore numbers and size.

Sample	Total Number of Pores	Biggest Pore (µm^3^)	80% of Pores Are Smaller Than (µm^3^)
AZ31	2768	633551	295
AZ61	2892	571775	252
AlMg3	3172	70620	388
